# Supplementary material for: Mucilaginibacter sp. Strain Metal(loid) and Antibiotic Resistance Isolated from Estuarine Soil Contaminated Mine Tailing from the Fundão Dam
Source: Genes (Basel). 2022 Jan 19;13(2):174. doi: 10.3390/genes13020174 (PMC8871858; doi:10.3390/genes13020174)
Supplement: Supplementary file 1 [file genes-13-00174-s001.zip › genes-1513269-supplementary.pdf]

Supplementary data

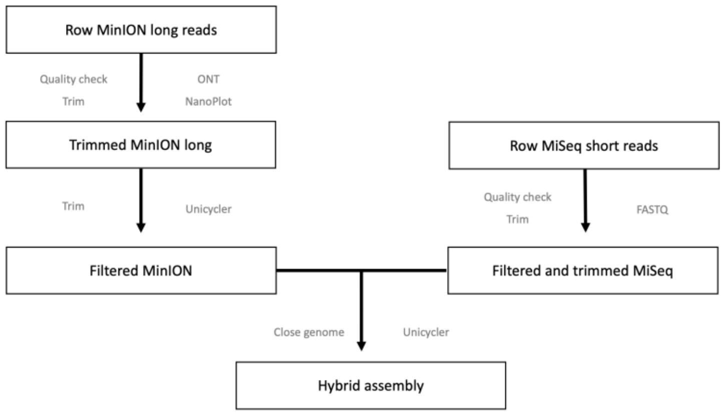

Figure S1. Bioinformatic workflow of the hybrid, MinION and MiSeq assembly.

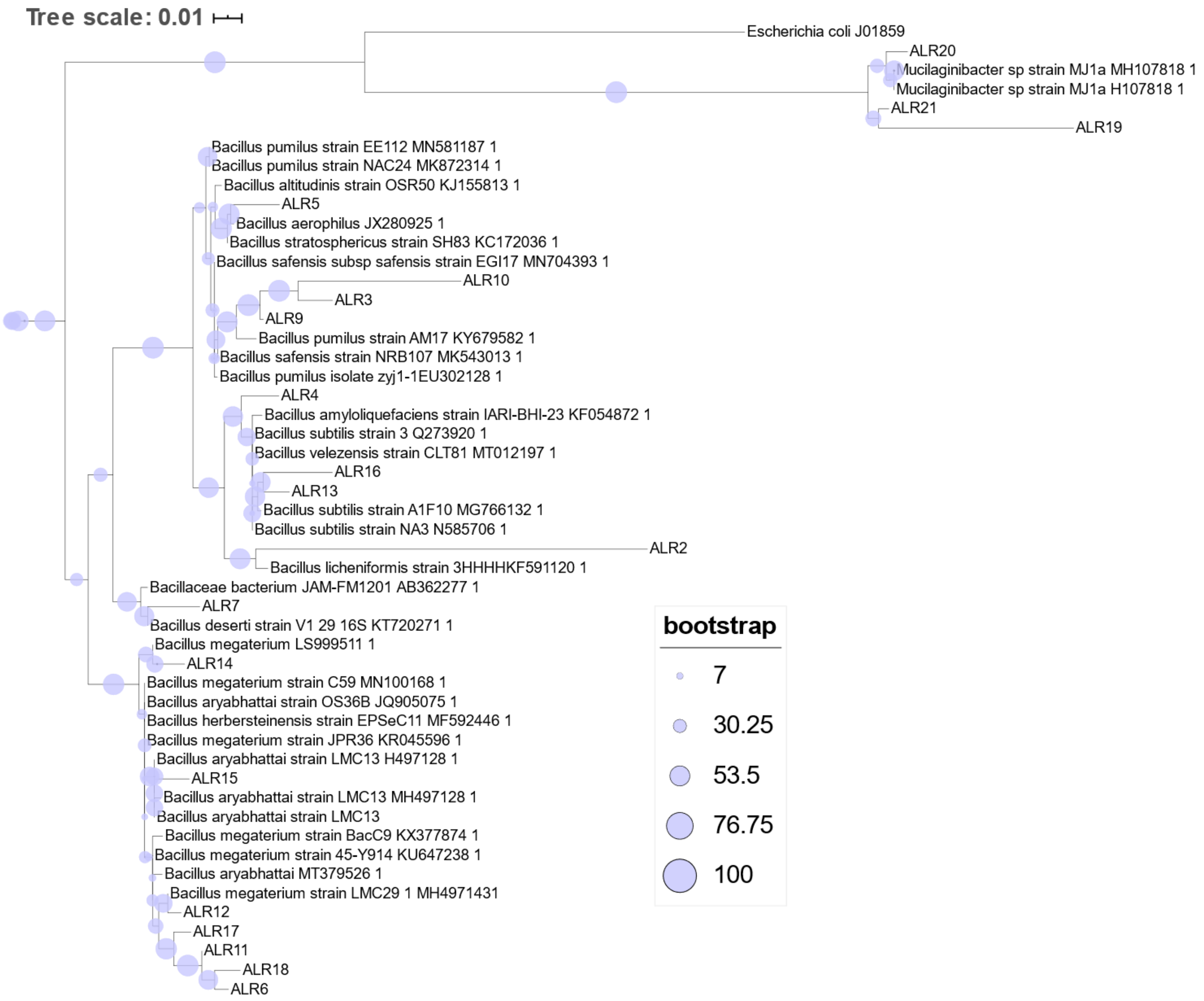

Figure S2. Neighbor-joining phylogenetic tree constructed based on the 16S ribosomal RNA (rRNA) gene sequences from bacteria isolated showing the phylogenetic relationships between all isolated strains including *Mucilaginibacter* sp. 21p. Values indicate percentages of identical branching in 100 bootstrapping. The sequence of Escherichia coli J01859 was used as an out-group.

Table S1: Direct submission of sequence data to GenBank

**Submission Number and Taxomonic**		
SUB8325601 ALR1	MW187503	*Bacillus* sp.
SUB8325601 ALR2	MW187504	*Bacillus licheniformis*
SUB8325601 ALR3	MW187505	*Bacillus pumilus*
SUB8325601 ALR4	MW187506	*Bacillus* sp.
SUB8325601 ALR5	MW187507	*Bacillus aerophilus*
SUB8325601 ALR6	MW187508	*Bacillus* sp.
SUB8325601 ALR7	MW187509	*Bacillus* sp.
SUB8325601 ALR9	MW187510	*Bacillus pumilus*
SUB8325601 ALR10	MW187511	*Bacillus pumilus*
SUB8325601 ALR11	MW187512	*Bacillus* sp.
SUB8325601 ALR12	MW187513	*Bacillus megaterium*
SUB8325601 ALR13	MW187514	*Bacillus subtilis*
SUB8325601 ALR14	MW187515	*Bacillus megaterium*
SUB8325601 ALR15	MW187516	*Bacillus aryabhattai*
SUB8325601 ALR16	MW187517	*Bacillus subtilis*
SUB8325601 ALR17	MW187518	*Bacillus* sp.
SUB8325601 ALR18	MW187519	*Bacillus* sp.
SUB8325601 ALR19	MW187520	*Mucilaginibacter* sp.
SUB8325601 ALR20	MW187521	*Mucilaginibacter* sp.
SUB8325601 ALR21	MW187522	*Mucilaginibacter* sp.
**SUB8286515 PRJNA667924**		**SAMN16392370 *Mucilaginibacter* sp. 21p**

Table S2. Chemical Analysis of the soil where the strains were selected.

**Sample**	**Total metal in mg kg^{-1}**												
	**Al**	**Cd**	**Co**	**Cr**	**Cu**	**Fe**	**Mn**	**Ni**	**P**	**Pb**	**Zn**	**pH**	**Eh**
Contaminated soil	7370	3.1	8.8	30.3	10.7	30396	459.8	10.1	387.4	13.6	48.4	6.5	370
Pristine Soil	1082	--	1.2	4,1	3.6	10489	50.0	2.3	--	4.8	2.4	--	--

* Data based on Gomes et al. 2017, study that collect samples 2 days before the disaster in the same that this study; Cd, pH and Eh was not informed.

Table S3. Average Nucleotide Identification (Blast algorithm).

	**Nome**	**ANIb [%]**	**Aligned [%]**	**Aligned [bp]**	**Total [bp]**	**RefSeq**
1	*Mucilaginibacter paludis*	69.38	36.33	1722017	4739655	NZ_CM001403.1
2	*Mucilaginibacter psychrotolerans*	71.83	46.90	2222846	4739655	NZ_SOZE00000000.1

3	*Mucilaginibacter phyllosphaerae*	73.76	51.95	2462155	4739655	NZ_SNQG00000000.1;
4	*Mucilaginibacter kameinonensis*	70.41	45.97	2178982	4739655	NZ_RCWZ00000000.1;
5	*Mucilaginibacter gotjawali*	69.80	38.06	1804141	4739655	NZ_AP017313.1
6	*Mucilaginibacter lappiensis*	70.50	44.25	2097240	4739655	NZ_FTMG00000000.1;
7	*Mucilaginibacter polytrichastri*	69.90	39.83	1887980	4739655	NZ_FPAM00000000.1
8	*Mucilaginibacter gossypii*	70.72	44.74	2120667	4739655	NZ_CP043449.1
9	*Mucilaginibacter hurinus*	70.08	32.92	1560174	4739655	NZ_QGDC00000000.1
10	*Mucilaginibacter corticis*	70.39	37.13	1759804	4739655	NZ_VLPK00000000.1
11	*Mucilaginibacter terrigena*	73.88	49.65	2353321	4739655	NZ_SEWG00000000.1;
12	*Mucilaginibacter limnophilus*	70.30	37.44	1774487	4739655	NZ_SACK00000000.1
13	*Mucilaginibacter endophyticus*	70.47	46.14	2186976	4739655	NZ_QPCG00000000.1
14	*Mucilaginibacter xinganensis*	70.21	39.86	1889277	4739655	NZ_CP022743.1
15	*Mucilaginibacter ginsenosidivorax*	70.59	43.98	2084666	4739655	NZ_CP042437.1
16	*Mucilaginibacter ginsenosidivorans*	69.54	36.84	1746029	4739655	NZ_CP042436.1
17	*Mucilaginibacter frigoritolerans*	70.02	39.00	1848280	4739655	NZ_VLLI00000000.1
18	*Mucilaginibacter gracilis*	69.62	33.99	1611240	4739655	NZ_RBKU00000000.1
19	*Mucilaginibacter rubeus*	70.39	46.65	2211131	4739655	NZ_CP043450.1
20	*Mucilaginibacter oryzae*	70.70	43.84	2077853	4739655	NZ_QGHA00000000.1
21	*Mucilaginibacter yixingensis*	70.14	33.37	1581560	4739655	NZ_QAOQ00000000.1
22	*Mucilaginibacter auburnensis*	70.12	33.74	1599015	4739655	NZ_PGFJ00000000.1
23	*Mucilaginibacter pedocola*	71.96	49.97	2368607	4739655	NZ_MBTF00000000.1
24	*Mucilaginibacter gossypiicola*	70.47	46.54	2205933	4739655	NZ_FOCL00000000.1;
25	*Mucilaginibacter mallensis*	70.20	38.57	1827886	4739655	NZ_LT629740.1
26	*Mucilaginibacter pineti*	70.66	44.07	2088850	4739655	NZ_FNAI00000000.1
27	*Mucilaginibacter boryungensis*	70.36	33.57	1639307	4882537	NZ_JADFFM010000001.1
28	*Mucilaginibacter glaciei*	69.84	35.18	1709210	4857968	NZ_JACWMX000000000.1
29	*Mucilaginibacter galii*	72.21	50.55	2322903	4595613	NZ_BMDO01000018.1
30	*Mucilaginibacter myungsuensis*	69.63	33.89	1723720	5086043	NZ_JADFFL000000000.1
31	*Mucilaginibacter pankratovii*	72.08	37.34	2378066	6368633	NZ_JACWMY000000000.1
32	*Mucilaginibacter rigui*	74.11	53.22	2506078	4708985	NZ_JACWMW000000000.1

Table S4. List of alleles that is not present in assembly *M. 21p* e according to Pirate.

Gene	Description	Frequency*
chonabc	Chondroitin sulfate ABC exolyase	1
abnA	Extracellular endo-alpha-(1->5)-L-arabinanase 1	1
nagAa	Naphthalene C2-salicylate 5-hydroxylase systems ferredoxin--NAD(P)(+)%2C reductase	1
narX	Nitrate/nitrite sensor protein NarX	1
nap	putative carboxylesterase nap	1
narS	putative sensor histidine kinase NarS	2
tnaA1	Tryptophanase 1	2
nasC	Assimilatory nitrate reductase catalytic subunit	2
phnA	Phosphonoacetate hydrolase	2
xynA	Endo-1%2C4-beta-xylanase A	2
nagJ	hypothetical protein	4

arnA	Bifunctional polymyxin resistance protein ArnA	6
nanR	HTH-type transcriptional repressor NanR	6
nanM	N-acetylneuraminate epimerase	7
napA	Periplasmic nitrate reductase	15
nasD	Nitrite reductase [NAD(P)H]	16
nasF	Uroporphyrinogen-III C-methyltransferase	18

*Comparative analyze of frequency of each specie contain respective gene.
